# Does previous abdominal surgery adversely affect perioperative and oncologic outcomes of laparoscopic radical cystectomy?

**DOI:** 10.1186/s12957-018-1317-6

**Published:** 2018-01-17

**Authors:** Xiaosong Wei, Jinjin Lu, Khurram Mutahir Siddiqui, Fan Li, Qianyuan Zhuang, Weimin Yang, Zhiquan Hu, Zhong Chen, Xiaodong Song, Shaogang Wang, Zhangqun Ye

**Affiliations:** 1 Department of Urology, Tongji Hospital, Tongji Medical College, Huazhong University of Science and Technology, No. 1095 Jiefang Ave, Wuhan, 430030 Hubei People’s Republic of China; 2 Department of Urology, First Affiliated Hospital of Zheng Zhou University, Zhengzhou, 450052 Henan People’s Republic of China; 3 Departments of Surgery (Urology), Western University, London Health Sciences Centre, London, ON N6A 5W9 Canada

**Keywords:** Bladder cancer, Radical cystectomy, Laparoscopic surgery, Previous abdominal surgery, Perioperative, Oncologic outcomes

## Abstract

**Background:**

Laparoscopic radical cystectomy (LRC) has been shown to have less estimated blood loss (EBL), transfusion rate, narcotic analgesic requirement, earlier return of bowel function, and shorter hospital stay. The aim of this study was to investigate the feasibility, peri-operative and oncologic outcomes of laparoscopic radical cystectomy (LRC) in patients with previous abdominal surgery (PAS).

**Methods:**

We retrospectively reviewed 243 patients undergoing open radical cystectomy (ORC) or LRC with bilateral pelvic lymph node dissection and urinary diversion or cutaneous ureterostomy at a single center from January 2010 to December 2015. Demographic parameters, intra-operative variables, peri-operative records, pathologic outcomes, and complication rate were reviewed to assess the impact of PAS on peri-operative and oncologic outcomes.

**Results:**

Patients in both ORC and LRC subgroups were homogeneous in terms of demography characteristics including age, gender, BMI, ASA score, and comorbidity. Estimated blood loss (EBL) was higher in patients with PAS undergoing ORC compared to those with no PAS (*P* = 0.008). However, there was no significant difference of EBL among patients undergoing LRC with or without PAS (*P* = 0.896). There was no statistical difference in peri-operative parameters and pathological outcomes. Patients with PAS undergoing ORC and ileal conduit had a higher vascular injury rate (*P* = 0.017). Comparing patients with PAS performed by LRC and ORC, the number of patients with the vascular injury was higher in ORC groups regardless of the type of diversion (ileal conduit, *P* = 0.001, cutaneous ureterostomy, *P* = 0.025). There is no significant difference in other complications.

**Conclusion:**

The presence of adhesions from PAS is not a contraindication to LRC. Patients with PAS may benefit from LRC with lower estimated blood loss, fewer transfusion rates, and vascular injuries. Furthermore, the overall oncologic outcomes and complication rate are similar between LRC and ORC patients with PAS.

## Background

Bladder cancer is the ninth most common cancer in the world. It was estimated that 20.1 per 100,000 new patients would be diagnosed with bladder cancer, and there would be 4.4 per 100.000 projected deaths from this disease annually [1]. Radical cystectomy (RC) provides excellent local control and long-term survival, for those with high-risk non-muscle invasive bladder cancer (NMIBC), such as T1G3 or muscle-invasive bladder cancer (MIBC). Laparoscopic-assisted surgery is a minimally invasive option to promote early recovery and reduce hospital stay with comparable mortality and morbidity. The overall high complication rate from open radical cystectomy (ORC) has compelled numbers of urologists to evaluate the technical feasibility and oncologic effectiveness of LRC [2–4]. Laparoscopic radical cystectomy (LRC) has been shown to have significantly less estimated blood loss (EBL), transfusion rate, narcotic analgesic requirement, earlier return of bowel function, and shorter hospital stay.

A number of individuals undergoing radical cystectomy have a history of previous abdominal surgical (PAS). To the best of our knowledge, adhesions have been reported in up to 95% cases after abdominal surgeries. PAS is usually considered to be a surgical challenge and many urologists tend to offer open surgery to these patients due to the fear that LRC may adversely affect the outcomes [2, 3, 5, 6]. Thus, PAS is considered as a relative contraindication for LRC. Studies with small numbers have documented the feasibility of LRC in patients with PAS [5, 7]. Proponents of LRC have shown that presence of adhesions is not a contraindication to LRC, and the overall complication rate and oncologic outcomes were similar between open and laparoscopic assisted groups [8–11]. While LRC has been criticized due to longer operative time and potential of higher risk for intra- and post-operative complications [5, 12], we interrogated the impact of ORC and LRC on intra-/post-operative and oncologic outcomes in bladder cancer patients with PAS.

## Methods

A retrospective chart review was performed for 243 patients undergoing radical cystectomy for MIBC or high-risk NMIBC urothelial carcinoma at the Department of Urology Tongji Hospital (China) from January 2010 to December 2015. Clinical data were split into two categories: radical cystectomy with the urinary diversion of the ileal conduit or cutaneous ureterostomy. Each category was further sub-divided into two subgroups with the open or laparoscopic procedure. The demographic parameters, peri-operative, pathological and oncologic outcomes, and intra-/post-operative complications were compared. PAS was defined as any surgical procedure that is potentially known to cause abdominal or pelvic adhesions. Patients who received neo-adjuvant chemotherapy were excluded for limited in quantity. This study was approved by the ethical board of Tongji Hospital (IRB ID: TJ-C20141219). All procedures performed are in accordance with the ethical principles of Declaration of Helsinki.

### Surgical procedure

The night before surgery, all RC patients were administered to bowel preparation with 50% magnesium sulfate and soapsuds enema. 1 h before surgery starting, patients sequentially accepted intravenous cefperazone-sulbactam 1.0 g and metronidazole 1.0 g for antibiotics prophylaxis.

The standard bilateral pelvic lymph node dissection (PLND) was performed in all patients to the same extent [13]. Open radical cystectomy (ORC) with ileal conduit (IC) or cutaneous ureterostomy (CU) was performed according to the conventional procedure [13, 14]. Laparoscopic radical cystectomy was performed by using the five ports technique described by Huang et al. [15].

Ileal conduit diversion was performed extracorporeally through a 6 cm supraumbilical midline incision in the LRC group. Laparoscopic cutaneous ureterostomy was performed as follows. The distal maneuverable part of ureter was mobilized from retroperitoneal space from an incision on the peritoneum. Then the bilateral ureters were grasped and pulled to the abdominal wall through the 10 mm trocar port on the right side of the lateral margin of the abdominal rectus. Pneumoperitoneum was deflated to minimize the distance to the abdominal wall. The stoma was then matured. All patients were placed on a standardized post radical cystectomy care plan.

### Outcomes measured

The demographic parameter records include age, gender, body mass index (BMI), surgery history, comorbidities, American Society of Anesthesiologists (ASA) scores, and primary/recurrence cancer. Operative variables included operative time, estimated blood loss (EBL), transfusion requirement of red blood cell, and fresh frozen plasma. EBL on the anesthesia note was estimated by anesthetists and was defined as the blood loss in aspirator and on the surgical gauze and sponge. Washing liquid had been excluded from the total volume by anesthetists after the surgical procedure. Peri-operative outcomes studied were compared in hemoglobin, time to return of liquid diet intake, time to drainage tube removal, and length of hospital stay. The clinical stage and pathological grade were recorded according to the TNM classification and the World Health Organization (WHO) system in 2004, respectively. Oncologic outcomes were assessed with surgical margin status and lymph node dissection numbers (LNDs). Intra-operative complications including vascular or bowel injury, and post-operative complications, systemic inflammatory response syndrome (SIRS), wound infection, wound dehiscence, prolonged ileus, intestinal anastomotic leakage, urinary anastomotic leakage, and deep venous thrombosis (DVT). All post-operative complications were also graded according to the Clavien-Dindo classification system in the first 90 days post-operatively.

### Statistical analysis

Differences in variables with a continuous normal distribution were analyzed by using the Student’s *t* test, while variables with continuous non-normal distribution were analyzed by non-parametric Mann-Whitney *U* test. Statistical analyses for categorical variables were performed using Pearson chi-square test or Fisher’s exact test. The logistic regression model was applied to correlate the real impact of various clinical and pathological variables’ affects on clinical and pathological outcomes. Statistical analysis was performed with SPSS 19.0 statistical software (IBM Inc., Armonk, NY, USA). All reported *P* values were statistical significance at *P* < 0.05.

## Results

### Demography

Radical cystectomy (RC) with ileal conduit (IC) diversion was performed in 132 patients, included 69 patients with ORC and 63 patients with LRC. One hundred and eleven patients underwent RC with cutaneous ureterostomy, including 73 patients with ORC and 38 patients with LRC. The demographic characteristics including age, gender, BMI, ASA score and comorbidity were comparable with no statistically significant difference as shown in Tables 1 and 2. However, in ORC groups, the number of patients with recurrence bladder cancer was higher in PAS group (ileal conduit, *P* = 0.042; ureterostomy, *P* = 0.020, respectively), but this difference was not significant in LRC groups (ileal conduit, *P* = 0.201, ureterostomy, *P* = 0.282, respectively). For patients undergoing RC with ureterostomy, the number of recurrence bladder cancer patients with PAS in ORC group was higher than LRC group (*P* = 0.043).

**Table 1:** Demography for patients with radical cystectomy and ileal conduit

	ORC (69)			LRC (63)			ORC vs. LRC with PAS
	PAS			PAS			
	No	Yes	*P* value	No	Yes	*P* value	*P* value
Demography	50	19		51	12		
Age	58.2 ± 9.0	54.6 ± 10.0	0.156*	59.9 ± 9.0	61.8 ± 9.9	0.538*	0.061*
Body mass index (BMI) (Kg/m^2^)	24.2 ± 3.8	26.0 ± 4.6	0.120*	24.6 ± 3.9	26.1 ± 2.5	0.211*	0.937*
Gender			1.000^#^			1.000^#^	1.000^#^
Male	43	17		45	11		
Female	7	2		6	1		
ASA score^a^	14		1.000^#^	45		0.201^#^	0.350^#^
1	1	1		3	0		
2	6	2		24	4		
3	3	1		9	5		
4	0	0					
Primary/recurrence tumor			0.042^#^			0.282^#^	1.000^#^
Primary	32	7		30	5		
Recurrence	18	12		21	7		

*Student’s *t* test was used for statistical analysis

^#^Chi-square test was used for statistical analysis

^a^ASA score was started applying in anesthesiology assessment from 2013. Only 14 cases in open and 45 cases in laparoscopic were evaluated by ASA, respectively

**Table 2:** Demography for patients with radical cystectomy and cutaneous ureterostomy

	ORC (73)			LRC (38)			ORC vs. LRC with PAS
	PAS			PAS			
	No	Yes	*P* value	No	Yes	*P* value	*P* value
Demography	48	25		27	11		
Age	69.7 ± 10.5	64.8 ± 13.5	0.054*	64.3 ± 7.7	63.4 ± 11.8	0.805*	0.881*
Body mass index (BMI) (Kg/m^2^)	22.6 ± 3.0	21.5 ± 3.3	0.181*	23.5 ± 3.6	23.2 ± 4.4	0.826*	0.210*
Gender			0.975^#^			0.872^#^	0.961^#^
Male	40	20		22	8		
Female	8	5		5	3		
ASA score^a^	30		0.566^#^	29		0.201^#^	0.413^#^
1	0	0		0	1		
2	10	2		15	3		
3	11	6		7	3		
4	1	0		0	0		
Primary/recurrence tumor			0.02^#^			0.538^#^	0.043^#^
Primary	31	9		15	9		
Recurrence	17	16		12	3		

*Student’s *t* test was used for statistical analysis

^#^Chi-square test was used for statistical analysis

^a^ASA score was started applying in anesthesiology assessment from 2013. Only 30 cases in open and 29 cases in laparoscopic were evaluated by ASA, respectively

### Peri-operative outcomes

For patients undergoing RC with IC, we found that the estimated blood loss and transfusion rates were higher in patients with previous abdominal surgery undergoing ORC compared to patient with LRC (*P* = 0.002, *P* = 0.005, respectively). We noted no statistical difference in pre-operative hemoglobin, serum albumin, operating time, time to liquid intake, drainage tube removal, and post-operative hospital stay by RC with ileal conduit intra- or inter-group comparison (Table 3). For patients undergoing RC with ureterostomy, we found that the estimated blood loss was also significant lesser in LRC group (*P* = 0.001), but no statistical difference has been found in transfusion rate (*P* = 0.214) (Table 4).

**Table 3:** Peri-operative information for patients with radical cystectomy and ileal conduit

	ORC (69)			LRC (63)			ORC vs. LRC with PAS
	PAS			PAS			
	No	Yes	*P* value	No	Yes	*P* value	*P* value
Pre-operation hemoglobin (HB) (g/L)	129.6 ± 16.5	129.1 ± 23.1	0.912*	129.9 ± 16.8	132.7 ± 17.4	0.606*	0.649*
Pre-operation serum albumin (g/L)	37.8 ± 2.9	37.8 ± 4.5	0.988*	38.8 ± 3.5	37.3 ± 3.2	0.173*	0.742*
Operating time (min)	397 ± 72	417 ± 67	0.297*	440 ± 79	435 ± 89	0.848*	0.522*
Estimated blood loss (ml)	1000 (788, 1700)	1600 (1200, 3000)	0.008**	500 (300, 1150)	550 (325, 900)	0.896**	0.002**
Transfusion no.	46	19	0.327#	34	7	0.738#	0.005#
Time to liquid intake (days)	6 (5, 7)	6 (5.75, 7.25)	0.799**	5.5 (5, 6)	5 (4.25, 5)	0.176**	0.096**
Time to drainage tube removal (days)	10.5 (7, 14)	10.5 (7.75, 21.25)	0.642**	9 (7, 12.75)	8.5 (7, 18.75)	0.986**	0.393**
Post-operation hospital stay (days)	17 (13, 22)	21.5 (14.75, 29.5)	0.185**	14.5 (13, 20)	15.5 (13, 34.25)	0.358**	0.340**

*Student’s *t* test was used for statistical analysis

**Mann-Whitney *U* test was used for statistical analysis

#Chi-square test was used for statistical analysis

**Table 4:** Peri-operative information for patients with radical cystectomy and cutaneous ureterostomy

	ORC (73)			LRC (38)			ORC vs. LRC with PAS
	PAS			PAS			
	No	Yes	*P* value	NO	Yes	*P* value	*P* value
Pre-operation hemoglobin (HB) (g/L)	116.9 ± 20.3	115.8 ± 17.7	0.820*	128.1 ± 17.2	122.4 ± 12.9	0.333*	0.273*
Pre-operation serum albumin (g/L)	35.1 ± 4.3	36.3 ± 4.5	0.247*	38.3 ± 3.2	35.9 ± 3.1	0.040*	0.736*
Operating time (min)	240 (210,308)	240 (210,285)	0.664**	285 (240,360)	308 (236,398)	0.246**	0.143**
Estimated blood loss (ml)	1000 (600,1850)	1100 (600,2000)	0.964**	350 (200,463)	450 (288,775)	0.163**	0.001**
Transfusion no.	46	21	0.172 #	12	7	0.283 #	0.214
Time to liquid intake (days)	3 (3,4.25)	4 (3,4)	0.148**	3 (2,5)	2 (2,5.5)	0.751**	0.751**
Time to drainage tube removal (days)	8 (6.75,9.25)	7 (7,13)	0.851**	10 (6.75,15)	10 (9,15.75)	0.961**	0.128**
Post-operation hospital stay (days)	13 (10.75,16.25)	13 (11,17)	0.379**	14.5(11.75, 18.25)	14(13,18.75)	0.604**	0.654**

*Student’s *t* test was used for statistical analysis

**Mann-Whitney *U* was used for statistical analysis

^#^Chi-square test was used for statistical analysis

### Pathologic outcomes

The histological diagnosis of all the patients in this study was urothelial carcinoma. The average positive surgical margin (PSM) rate was 9%. And the overall median number of lymph node (LN) yield was 10. The TNM stage and pathological grade were evenly distributed and illustrated in Tables 5 and 6, respectively.

**Table 5:** Pathologic parameters for patients with radical cystectomy and ileal conduit

	ORC (69)			LRC (63)			ORC vs. LRC with PAS
	PAS			PAS			
	No	Yes	*P* value	No	Yes	*P* value	*P* value
T stage			0.517^#^			0.239^#^	0.360^#^
T1, Tis	18	5		23	7		
T2	15	5		17	2		
T3	13	5		9	1		
T4	4	4		2	2		
N stage			0.389^#^			0.400^#^	0.555^#^
N0	44	16		46	10		
N1	3	0		2	1		
N2	3	3		3	1		
N3	0	0		0	0		
Tumor grade (WHO 2004)			0.446^#^			0.538^#^	0.620^#^
Low grade	18	5		14	5		
High grade	32	14		37	7		
Positive surgical margin (PSM)	4	2	1.000^#^	2	1	1.000^#^	1.000^#^
Lymph node dissection (LND)	10.6 ± 3.9	11.9 ± 4.6	0.279*	11.0 ± 2.8	11.9 ± 3.9	0.473*	0.777*

^#^Chi-square test was used for statistical analysis

*Student’s *t* test was used for statistical analysis

**Table 6:** Pathologic parameters for patients with radical cystectomy and cutaneous ureterostomy

	ORC (73)			LRC (38)			ORC vs. LRC with PAS
	PAS			PAS			
	No	Yes	*P* value	No	Yes	*P* value	*P* value
T stage			0.321^#^			0.744^#^	0.788^#^
T1, Tis	11	8		8	5		
T2	22	7		6	2		
T3	7	7		9	2		
T4	8	3		4	2		
N stage			0.865^#^			0.469^#^	1.000^#^
N0	42	24		21	11		
N1	3	0		3	0		
N2	2	1		3	0		
N3	1	0		0	0		
Tumor grade (WHO 2004)			0.639^#^			1.000^#^	1.000^#^
Low grade	14	6		6	2		
High grade	34	19		21	9		
Positive surgical margin (PSM)	5	3	1.000^#^	3	2	0.615^#^	1.000^#^
Lymph node dissection (LND)	9.1 ± 3.5	10.4 ± 3.6	0.14*	10.1 ± 2.4	11.3 ± 3.1	0.253*	0.403*

*Student’s *t* test was used for statistical analysis

#Chi-square test was used for statistical analysis

### Complications

We performed a logistic regression model to control various clinical and pathological variables, including gender, PAS, ORC or LRC, primary/recurrence, TNM staging, pathologic grade, and urinary diversion. The PAS has not been found to be an adverse variable to predict the post-operative complication (*P* = 0.107). In the same analysis, the urinary diversion approach (ileal conducts vs. cutaneous ureterostomies) was an adverse predictor to the intra-operative vascular injury (*P* = 0.017) and post-operation complication rate (*P* < 0.001). By applying Pearson chi-square tests, no significant difference between abdominal and pelvic PAS on intra-operative vascular injury (*P* = 0.594) or post-operative complication (*P* = 0.342) had been noted in our analysis. ORC was also been found to be a variable to predict intra-operative complication concurrence rate. The vascular injury rate was found to be higher in patients treated by ORC (*P* = 0.017). Similarly, the vascular complication was significantly higher in patients with PAS undergoing ORC compared to LRC (*P* = 0.001). Post-operative complications were graded according to Clavien-Dindo classification system. The detailed comparison is shown in Tables 7 and 8.

**Table 7:** Complication for patients with radical cystectomy and ileal conduit

	ORC (69)			LRC (63)			ORC vs. LRC with PAS
	PAS			PAS			
	No	Yes	*P* value	No	Yes	*P* value	*P* value
Clavien-Dindo classification			0.191^#^			0.060^#^	0.445^#^
I	2	2		2	1		
II	3	2		7	1		
IIIa	3	1		1	3		
IIIb	2	3		2	0		
IVa	0	0		0	0		
IVb	0	0		0	0		
V	0	0		0	0		
Vascular injury	31	18	0.017^#^	19	4	1.000^#^	0.001^#^
Bowel injury	0	1	0.275^#^	0	0	–	1.000^#^
SIRS	4	2	1.000^#^	1	1	0.828^#^	1.000^#^
Wound infection	5	5	0.181^#^	5	4	0.102^#^	0.990^#^
Wound dehiscence (need secondary suture)	5	3	0.803^#^	3	3	0.138^#^	0.868^#^
Intestinal obstruction, ileus	2	2	0.646^#^	5	1	1.000^#^	1.000^#^
Intestinal leakage	0	1	0.275^#^	0	0	–	1.000^#^
Urine leakage	1	0	1.000^#^	0	0	–	–
Thrombosis	1	0	1.000^#^	1	1	0.828^#^	0.387^#^

^#^Chi-square test was used for statistical analysis

**Table 8:** Complication for patients with radical cystectomy and cutaneous ureterostomy

	ORC (73)			LRC (38)			ORC vs. LRC with PAS
	PAS			PAS			
	No	Yes	*P* value	No	Yes	*P* value	*P* value
Clavien-Dindo classification			0.541^#^			0.762^#^	1.000^#^
I	1	0		0	0		
II	0	1		1	1		
IIIa	1	2		0	0		
IIIb	1	0		2	0		
IVa	0	0		0	0		
IVb	0	0		0	0		
V	1	0		0	0		
Vascular injury	30	18	0.417^#^	4	3	0.662^#^	0.025^#^
Bowel injury	0	0	–	0	0	–	
SIRS	0	1	0.342^#^	0	0	–	1.000^#^
Wound infection	3	1	1.000^#^	1	0	1.000^#^	1.000^#^
Wound dehiscence (need secondary suture)	2	1	1.000^#^	1	0	1.000^#^	1.000^#^
Intestinal obstruction, ileus	0	2	0.114^#^	1	1	1.000^#^	1.000^#^
Intestinal leakage	0	0	–	0	0	–	–
Urine leakage	0	0	–	1	0	1.000^#^	–
Thrombosis	0	1	0.342^#^	0	0	–	1.000^#^

^#^Chi-square test was used for statistical analysis

## Discussion

In the last two decades, with the promise of reducing peri-operative morbidity and improving oncologic and functional outcomes, laparoscopic surgery has made significant inroads in oncologic surgery including high-risk bladder cancer [4, 16]. Decreased EBL, lesser transfusions, rapid return of bowel function, and shorter hospital stay are proven benefits [9, 11].

Intra-abdominal and pelvic adhesions are frequently encountered in patients with PAS. Numbers of studies have suggested that abdominal and pelvic adhesions are associated with intra-operative vascular and bowel injuries [17, 18]. This prohibits numbers of urologists to offer laparoscopic surgery due to the fear of causing additional injuries. In the present study, 28% patients in our study have PAS history. The demographic characteristics of all our subgroups were homogenous, and history of PAS did not restrict the decision to offer open or laparoscopic surgery at our center.

PAS pose two significant challenges; the first hurdle is the entry into peritoneal space. To safely proceed with the intended LRC procedures, surgeons might lysis adhesions before trocar placement. Poorly placed trocar may preclude efficient completion of the LRC, and processing around adhesions may potentially lead to visceral or vascular injury. Upon entering the peritoneal space, the adhesions require taking down to accurately identify anatomical landmarks and progress with the dissection. Our confidence in this technique is reflected by the fact that no open conversion was needed in our LRC cases.

Operative time, time to drainage tube removal, and length of hospital stay in patients undergoing LRC were not only comparable to our ORC cohort but also similar to other published report on LRC without previous PAS [12, 19]. In our series, we report a lower EBL and transfusion rate in patients with PAS undergoing LRC compared to ORC subgroups. Peri-operative transfusion is an independent predictor of decreased overall survival in patients with solid organ malignancies. Based on a large cohort study, Linder et al. noted that due to the potential immunosuppressive effect of red blood cell transfusion, perioperative blood transfusion (PBT) is associated with significantly increased risks of cancer recurrence and mortality following radical cystectomy [20]. Thus, bladder cancer patients with PAS may benefit from LRC by reducing transfusion rate. Intriguingly, by applying different statistic analysis, a controversial conclusion was concluded by Morgan et al. They contributed the paradox to inadequately and inaccurately control for relevant confounding variables. Such variables included increased age, surgical complexity, adverse tumor pathology, and possibly surgical expertise [21]. The authors emphasized that elucidating the association between PBT and prognosis should provide additional statistical modeling and further scrutiny. Unfortunately, randomized, prospective, long-term survival has not been provided in our study. In our logistic regression model, the urinary diversion approach was an adverse predictor to the intra-operative and post-operation complication rate. Thus, evaluating LRC and ORC with the urinary diversion of the ileal conduit and cutaneous ureterostomy separately made us better understanding the effect of PAS on post-operative outcomes. In this study, PAS did not adversely affect the early recovery of bowel function and return to normal activity for patients with LRC.

Post-operative hospital stay is a crude indicator of the recovery after surgery [22]. There are several biases which may affect this outcome, social norms, reimbursement models and availability of a reliable rehabilitative system are import considerations which cannot be ignored. Thus, the hospital stay may not always be considered as a parameter for post-operative recovery. The differences in the healthcare system between China and western countries have been noted before [2]. Regardless of fitness for discharge, almost all patients from subgroups in this study insisted on staying until drainage tubes and sutures were removed.

The ability of LRC to obtain the same quality of oncologic control as ORC has been questioned in several previous studies. Lymph node dissection number (LNDs) and positive surgical margin (PSM) status are two independent predictive factors for oncologic outcomes. Several studies have taken LNDs yield as an indicator of surgical quality with RC [23–25]. Previous studies documented that laparoscopic pelvic lymph node dissection may be hindered by the presence of abdominal adhesions [24, 26]. However, regardless fewer lymph nodes retrieved from the patients with PAS, these studies also noted that the lymph node dissection may still be carried out safely and reliably [27]. Herr et al. have described 10 to 14 lymph nodes dissected as an oncologic benchmark for RC [28]. This standard has been achieved in our cohort, with a median LNDs retrieved of 10. We acknowledge that the nodes removed in the pathological finding are under the mean of most of the series published. We attribute this issue to the factors below: (1) we regularly performed standard bilateral pelvic lymphadenectomy, but we rarely performed extended or super-extended PLND. This may significantly decrease the number of retrieved nodes, (2) during the PLND procedure, en bloc technique were preferentially performed on the patients who were possible. However, published studies had noted that more lymph nodes could be indentified in the pathological analysis by separated submission rather than en bloc submission [29]. Furthermore, we found no statistically significant difference in the node count among the two groups in general and those with or without PAS.

PSM has been reported as a significant predictor of recurrence and cancer-specific death. Zeng et al. noted that LRC achieved similar peri-operative outcomes to ORC without compromising oncologic or pathologic outcomes [2]. In our previous publication, LRC achieved an identical prognosis to ORC in terms of local recurrence and cancer-free survival [30]. In this study, for patients with PAS, LRC did not affect the positive margin rate compared to ORC. Herr et al. had described PSM rate of less than 10% as another benchmark [28]. However, some other studies had reported a PSM of less than 5% [31]. We acknowledge that our present PSM rate of 9% was higher than desirable. However, to our knowledge, none of the studies above had taken the pathological stage into consideration while calculating PSM rate. It should be noted that 34% patients (82 cases) were harboring locally advanced disease (53 had pT3 and 29 had pT4a) while none of the T1-T2 stage patients had PSM. Dotan et al. also noted that patients with organ confined disease were less likely to have PSM while tumor infiltration may be the main limitation in achieving a negative margin in locally advanced disease [32].

Several studies have demonstrated that a significantly higher frequency of complications after LRC in patients with PAS. Columbo et al. found a 48% complication rate in LRC patients, which made LRC an independent risk factor for peri-operative complications in patients with PAS [33]. Our overall complication rate was 18%, and there was no Clavien grade IV complication. Furthermore, for patients with PAS or without PAS, LRC did not adversely increase the complication rate compared to ORC subgroups. Zeng et al. reported a higher complication rate in elder patients undergoing LRC [2]. Several authors have previously determined age to be a significant risk factor for RC post-operative complications. In our center, the age of patients underwent RC ranged from 34 to 88 while the average age was 62, slightly less than other previous studies, which may explain why we have a lower post-operative complication rate. Because of higher comorbidities, senior patients should be carefully screened with stricter discretion and counseled about their risk of obstacles after surgery. Albisinni et al. reported re-operation rate of 12% in the first 30 days after LRC [24]. In our series, the reoperation rate was less than 8% patients in the first 90 days.

The limitations of the current studies include the biases associated with a retrospective study. Although a randomized, prospective study with long-term follow-up would be better to assess the effect of PAS on LRC, such a study is practically very difficult to conduct.

## Conclusions

The results of this study demonstrate that the oncologic efficacy and post-operative complications and morbidity rate are similar between patients with PAS undergoing LRC or ORC. The presence of adhesions from PAS is not a contraindication to LRC. Furthermore, patients with PAS may additionally benefit from LRC, due to lesser blood loss, transfusion rate, and intra-operative vascular injury rate.

## Abbreviations

ASA: American Society of Anesthesiologists
BMI: Body mass index
CU: Cutaneous ureterostomy
DVT: Deep venous thrombosis
EBL: Estimated blood loss
IC: Ileal conduit
LN: Lymph node
LNDs: Lymph node dissection number
LRC: Laparoscopic radical cystectomy
MIBC: Muscle-invasive bladder cancer
NMIBC: Non-muscle invasive bladder cancer
ORC: Open radical cystectomy
PAS: Previous abdominal surgery
PLND: Pelvic lymph node dissection
PSM: Positive surgical margin
RC: Radical cystectomy
SIRS: Systemic inflammatory response syndrome
WHO: World Health Organization
